# Mouse genome engineering uncovers 18 genes dispensable for male reproduction

**DOI:** 10.1111/andr.70088

**Published:** 2025-06-26

**Authors:** Hsin‐Yi Chang, Yonggang Lu, Kaito Yamamoto, Jiang Sun, Keisuke Shimada, Yuki Hiradate, Yoshitaka Fujihara, Masahito Ikawa

**Affiliations:** ^1^ Graduate School of Medicine The University of Osaka Suita Osaka Japan; ^2^ Department of Experimental Genome Research, Research Institute for Microbial Diseases The University of Osaka Suita Osaka Japan; ^3^ Premium Research Institute for Human Metaverse Medicine (WPI‐PRIMe) The University of Osaka Suita Osaka Japan; ^4^ Department of Advanced Medical Technologies National Cerebral and Cardiovascular Center Suita Osaka Japan; ^5^ The Institute of Medical Science The University of Tokyo Minato Tokyo Japan; ^6^ Center for Infectious Disease Education and Research (CiDER) The University of Osaka Suita Osaka Japan; ^7^ Center for Advanced Modalities and DDS (CAMaD) The University of Osaka Suita Osaka Japan

**Keywords:** CRISPR/Cas9, fertilization, male fertility, spermatogenesis, sperm morphology, sperm motility

## Abstract

**Background:**

Male infertility is an intricate multifactorial disease involving the interplay between genetic and environmental factors. Genetic anomalies account for more than 15% of all male infertility cases; however, diagnosing them exhibits enormous challenges due to variable symptomatic presentations and limited knowledge of gene functions. Therefore, a thorough investigation into gene regulatory networks underlying male reproduction is demanded to improve patient counseling and infertility treatment.

**Objective:**

In this study, we aimed to identify testis‐expressed genes essential for male fertility.

**Methods:**

We searched public databases, such as the National Center for Biotechnology Information (NCBI), Ensembl genome browser, the Human Protein Atlas (HPA), and the Mammalian Reproductive Genetics Database V2 (MRGDv2), to identify genes predominantly expressed in male reproductive tissues. Genetically engineered mouse lines lacking individual genes of interest were generated using either targeted gene replacement or the CRISPR/Cas9 system. To determine the gene functions, we analyzed fertility, testis weight, testis and epididymis histology, and sperm motility and morphology in adult knockout (KO) male mice.

**Results:**

Through the in silico screen, we identified 18 testis‐expressed genes, including coiled‐coil domain containing 182 (*Ccdc182*), EF‐hand calcium‐binding domain 15 (*Efcab15*), family with sequence similarity 187, member B (*Fam187b*), family with sequence similarity 24, member A (*Fam24a*), family with sequence similarity 24, member B (*Fam24b*), glial cell line derived neurotrophic factor family receptor alpha 2 (*Gfra2*), GLI pathogenesis‐related 1 like 1, 2, and 3 (*Glipr1l1‐3*), interleukin 3 (*Il3*), IZUMO family member 4 (*Izumo4*), peptidyl–prolyl cis/trans isomerase, NIMA‐interacting 1, retrogene 1 (*Pin1rt1*), solute carrier family 22 (organic cation transporter), member 16 (*Slc22a16*), sperm microtubule inner protein 2 (*Spmip2*), testis expressed 51 (*Tex51*), transmembrane and coiled‐coil domains 2 (*Tmco2*), and tripartite motif family‐like 1 and 2 (*Triml1*/*2*). The KO males displayed no obvious health problems, and normal mating behavior, fecundity, testis and epididymis histology, and sperm morphology and motility.

**Discussion and Conclusion:**

Our findings indicate that these 18 testis‐expressed genes are individually dispensable for male reproduction in mice. Disseminating such genes would promote our understanding of male reproduction and expedite the discovery of novel key male factors. Although we anticipate that mutations in these genes may not impair fertility in men, their enrichment in male germ cells makes them potential biomarkers for sperm count, quality, and morphological anomalies.

## 1 INTRODUCTION

A recent report by the World Health Organization estimated that one in six people experience infertility at certain periods in their lives.^1^ Among couples unable to conceive, approximately 50% of cases are partially or wholly attributed to male infertility.^2^, ^3^, ^4^, ^5^, ^6^, ^7^

Recent advances in next‐generation sequencing (NGS) have provided profound insights into the genetics of male infertility. Whole‐exome and whole‐genome sequencing have enabled comprehensive, high‐throughput identification of causative genetic variants for aberrant sperm formation, morphogenesis, and functionality.^8^, ^9^, ^10^, ^11^, ^12^, ^13^, ^14^ However, defining genotype–phenotype correlations remains challenging, as the majority of mutations detected by NGS have unknown significance for reproductive health.^15^

With the emergence of CRISPR/Cas9‐mediated genome engineering in mice, precise targeting of genes highly expressed in the gonads has become remarkably time and cost effective.^16^, ^17^, ^18^ Such functional genetics approaches have allowed the discovery of numerous molecules essential for reproductive success. Typical examples include *Pdcl2*,^19^ *Adad2*,^20^ *Tsks*,^21^ *Mettl16*,^22^ and *Ccer1*,^23^ which regulate sperm formation and differentiation; *Nell2*,^24^ *Nicol*,^25^ and *Ros1* ^26^ that coordinate lumicrine signaling essential for epididymal and sperm maturation; and *Izumo1*,^27^ *Spaca6*, *Sof1*, *Tmem95*,^28^ *Fimp*,^29^ *Dcst1/2*,^30^ and *Tmem81*,^31^ which are involved in sperm–egg plasma membrane binding and/or fusion. Notwithstanding substantial progress in the field, the etiology of genetically derived male infertility remains elusive, highlighting the need for continued efforts to uncover novel key male factors.

In this study, we identified 18 testis‐expressed genes through an in silico screen of public databases. To interrogate their roles in the male reproductive system, we generated knockout (KO) mouse lines by CRISPR/Cas9 or the conventional gene replacement method. Comprehensive phenotypic analyses on testis and epididymis histology, sperm morphology and motility, and male fecundity revealed that these genes are individually dispensable for male fertility in mice. Our findings imply potential functional redundancy or compensatory mechanisms within the gene regulatory networks governing male reproductive function.

## 2 MATERIALS AND METHODS

### 2.1 Animals

All experiments were approved by the Institutional Animal Care and Use Committees at The University of Osaka. B6D2F1 [hereafter referred to as wild‐type (WT)] and the Institute of Cancer Research (ICR) mice were procured from Japan SLC. KO mice were generated on the genetic background of B6D2 and maintained under specific‐pathogen‐free conditions with a 12‐h light/dark cycle and ad libitum feeding. Frozen spermatozoa of heterozygous KO mice have been deposited to RIKEN BioResource Research Center (BRC; web.brc.riken.jp/en) and Center for Animal Resources and Development (CARD) at Kumamoto University (card.medic.kumamoto‐u.ac.jp/card/English). The RIKEN BRC and CARD IDs for the deposited mouse lines are listed in Table S1.

### 2.2 Gene expression analyses

The expression profiles of each gene in mouse tissues and spermatogenic cells were retrieved from Mammalian Reproductive Genetics Database V2 (MRGDv2; orit.research.bcm.edu/MRGDv2)^32^ and a previously published single‐cell RNA sequencing dataset,^33^ respectively.

### 2.3 Phylogenetic analyses

Phylogenetic trees depicting gene conservation across species were obtained from TreeFam (treefam.org^34^; Figure S1A–I). Alternatively, the orthologous sequences of each protein were acquired from Ensembl (asia.ensembl.org), aligned using the ClustalW algorithm,^35^ and converted into phylogenetic trees with GENETYX Ver.11 (Nihon Server; Figure S1J–P).

### 2.4 Generation of KO mice by CRISPR/Cas9

*Ccdc182*, *Fam187b*, *Fam24a*, *Fam24b*, *Gfra2*, *Glipr1l1‐3*, *Il3*, *Izumo4*, *Pin1rt1*, *Slc22a16*, *Spmip2*, *Tex51*, *Tmco2*, and *Triml1/Triml2* KO mouse lines were generated by the CRISPR/Cas9 system. Two single guide RNAs (sgRNAs) targeting the 5′ and 3′ regions of each gene were designed by using webtools including CRISPRdirect (crispr.dbcls.jp),^36^ Benchling (benchling.com), and CRISPOR (crispor.tefor.net^37^; Table S2). WT females were intraperitoneally injected with 0.1 mL CARD HyperOva (Kyudo) and 5 IU human chorionic gonadotropin (hCG; Aska Animal Health) at noon, 48 h apart. Upon hCG administration, the female mice were individually caged with a WT male mouse. After 20 h, fertilized eggs were extracted from the oviductal ampulla of the superovulated females and treated with 330 µg/mL hyaluronidase (Sigma‐Aldrich) to remove the cumulus cells. The zygotes with two pronuclei were batch electroporated in Opti‐MEM (Thermo Fisher Scientific) containing CRISPR RNA (crRNA; Integrated DNA Technologies), trans‐activating crRNA (tracrRNA; Integrated DNA Technologies), and Cas9 (Thermo Fisher Scientific) ribonucleoprotein complex using a NEPA21 Super Electroporator (Nepa Gene). The electroporated zygotes were cultured in Potassium Simplex Optimized Medium (KSOM) until two‐cell stage and were transplanted into the oviductal ampulla of 0.5‐day post‐coitum pseudopregnant ICR female mice. Offspring were naturally delivered or obtained through cesarean section 19‐day post‐transplantation and genotyped by polymerase chain reaction (PCR) using primers enumerated in Table S3. The precise deletion patterns were determined by Sanger sequencing of the PCR amplicons representing the KO alleles.

### 2.5 Generation of *Efcab15* KO mice

*Efcab15* KO mice were generated by the conventional gene replacement method. Briefly, a 2.9 kb short and a 5.7 kb long homology arm were cloned and inserted into a pNT1.1 vector.^38^ To disrupt *Efcab15*, the targeting vector was linearized by enzymatic digestion with ClaI (New England Biolabs) and electroporated into EGR‐G101 embryonic stem cells (ESCs).^39^ The second to the ninth exons of *Efcab15* were replaced with a thymidine kinase (tk) expression cassette for negative selection and a neomycin resistance cassette (neo^r^) flanked by flippase recognition target (FRT) sites (Figure S2). ESC clones harboring homologous recombinations were selected by 150 µg/mL G418 and 2 µM ganciclovir, validated by genomic PCR, and microinjected into eight‐cell ICR embryos by a piezo‐driven micromanipulator (PRIME TECH). The injected embryos were cultured overnight in KSOM and transplanted into the uterine horns of 2.5‐day pseudopregnant ICR females. The chimeric male offspring obtained from the recipient mice were paired with WT females, and the resultant offspring were genotyped by PCR.

### 2.6 Fertility analyses of KO mice

Sexually mature (8–20 weeks old) WT and KO male mice were caged individually with one to three 6‐week‐old WT female mice for at least 8 weeks. After the mating period, the males were removed from the cages and the female mice were kept for additional 3 weeks to deliver the final litters. Copulatory plugs were recorded every morning and the numbers of pups were counted at birth. To analyze the fertility of *Gfra2* KO females, two 6‐week‐old KO females were caged with an 8‐ to 10‐week‐old WT male for 10 weeks. The females were then maintained for 3 more weeks to record the final litters.

### 2.7 Analyses of testis and epididymis histology

Testis and epididymis dissected from adult WT and KO males were fixed in Bouin's solution (Polysciences) at 4°C for overnight, embedded in paraffin wax, and cut into 5 µm thin sections using a Microm HM 325 Rotary Microtome (Thermo Fisher Scientific). The paraffin sections were rehydrated, stained with periodic acid (Nacalai Tesque) and Schiff's reagent (Wako), counterstained with Mayer's hematoxylin solution (Wako), mounted with Entellan™ new rapid mounting medium for microscopy (Merck) and imaged under an Olympus BX53 microscope equipped with an Olympus DP74 color camera (Evident).

### 2.8 Analyses of sperm motility and morphology

An incision was made at the tip of cauda epididymis, and spermatozoa were gently squeezed out and dispersed in the Toyoda, Yokoyama, and Hoshi (TYH) medium, followed by incubation at 37°C, 5% CO_2_. Motility parameters of non‐capacitated and capacitated spermatozoa were analyzed at 10 and 120 min post‐incubation, respectively, using the CEROS II sperm analysis system (Hamilton Thorne Biosciences). Sperm morphology was observed and captured under an Olympus BX53 microscope equipped with an Olympus DP74 color camera.

### 2.9 Statistical analysis

Statistical differences were evaluated by the two‐tailed Welch's *t*‐test using Microsoft Excel 2024 or by multiple Mann–Whitney tests using GraphPad Prism 9. Significance was attributed to *p* values below 0.05. The data are presented as mean ± standard deviation (SD).

## 3 RESULTS

### 3.1 In silico screen and expression analyses of testis‐expressed genes

**FIGURE 1:**
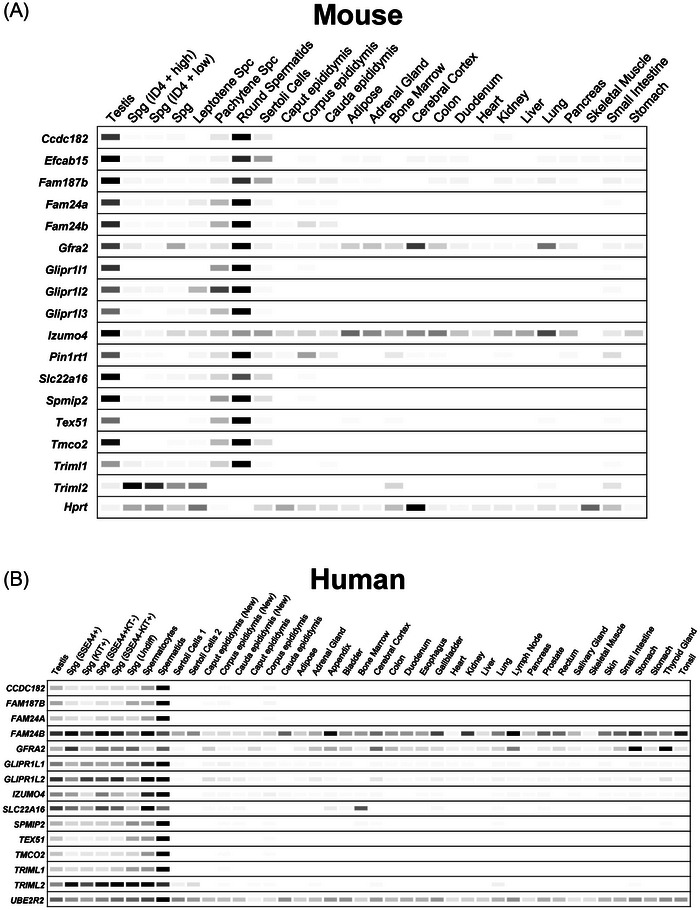
Tissue expression patterns of the candidate genes in mice and humans. (A) Digital polymerase chain reaction (PCR) of target genes in mouse tissues and cells. Band intensity represents the average transcripts per million (TPM). The expression of the housekeeping gene *Hprt* is shown as a positive control. (B) Digital PCR of target genes in human tissues and cells. The expression of the housekeeping gene *UBE2R2* is shown as a positive control. Spc, spermatocytes; Spg, spermatogonia.

Through a preliminary search of the National Center for Biotechnology Information (NCBI; ncbi.nlm.nih.gov/gene) and the Mouse Genome Informatics (MGI; informatics.jax.org) databases and a thorough literature review, we identified 18 evolutionarily conserved, testis‐expressed genes, *Ccdc182*, *Efcab15*, *Fam187b*, *Fam24a*, *Fam24b*, *Gfra2*, *Glipr1l1*, *Glipr1l2*, *Glipr1l3*, *Il3*, *Izumo4*, *Pin1rt1*, *Slc22a16*, *Spmip2*, *Tex51*, *Tmco2*, *Triml1*, and *Triml2*, whose physiological functions had not been reported in mice. According to the Encyclopedia of DNA Elements (ENCODE) transcriptome database (encodeproject.org) and MRGDv2,^32^ *Ccdc182*, *Fam187b*, *Fam24a*, *Fam24b*, *Gfra2*, *Glipr1l1*, *Glipr1l2*, *Izumo4*, *Slc22a16*, *Spmip2*, *Tex51*, *Tmco2*, *Triml1*, and *Triml2* are highly expressed in mouse and human testis and epididymis (Figure 1). Notably, *Fam24b* exhibits predominant expression in mouse testis but is ubiquitously expressed among human tissues and organs (Figure 1). *Efcab15* is a protein‐coding gene highly expressed in mouse testis (Figure 1A), while in humans, it is annotated as a pseudogene, *EFCAB15P* (NCBI gene identifier: 118568824). *Il3* shows biased expression in mouse testis (data retrieved from Expression Atlas; ebi.ac.uk/gxa; Figure S3A), whereas its human ortholog is expressed in testis, epididymis, and bone marrow (data retrieved from Human Protein Atlas [HPA]; proteinatlas.org; Figure S3B). *Pin1rt1* is identified as a retrogene of *Pin1*, highly expressed in mouse testis and epididymis (Figure 1A). Phylogenetic analyses indicated that several candidate genes, such as *Slc22a16* and *Tex51*, are highly conserved among Eukaryota, whereas the remainders, such as *Ccdc182*, *Fam24a*, *Fam24b*, *Gfra2*, *Glipr1l2*, *Il3*, and *Tmco2* exhibit limited conservation across Mammalia (Figure S1). Notably, *Glipr1l3* and *Pin1rt1* are only found in mice.

### 3.2 Generation of KO mice

KO mouse lines were generated to investigate the physiological functions of the 18 testis‐expressed genes in the male reproductive system. All genes were deleted using CRISPR/Cas9, except for *Efcab15*, which was disrupted by the conventional gene replacement method (see **Materials and Methods** section). Given that *Triml1* and *Triml2* are neighboring genes with considerable sequence homology (Figure S4A,B), we created a KO model lacking both genes. All relevant information about the mouse lines, including the sequences of crRNA protospacers and mutant alleles, embryo implantation outcomes, genome editing efficiency, and the primer sets and PCR conditions for genotyping, is presented in Tables S2–S4. The KO strategies of *Fam24a*, *Gfra2*, *Il3*, *Triml1/2*, and *Spmip2* are shown in Figure S5.

### 3.3 Fertility tests of KO mice

For each mouse line, three sexually mature KO males were individually caged with one to three WT females for at least 8 weeks. The KO male mice sired offspring with average litter sizes comparable to those of WT males (Table 1). Furthermore, we bred heterozygous KO males with homozygous KO females to maintain the mouse lines and observed no obvious reduction in the fecundity of KO females. A fertility test was conducted for *Gfra2* KO females, where three WT males were individually housed with two KO females for 10 weeks. The resultant litter size is comparable to that of WT mice (Table 1).

**TABLE 1:** Fertility tests of wild‐type (WT) and knockout (KO) male mice.

	Genotype	No. of mice analyzed	No. of pups	No. of litters	Mating period (weeks)	Average litter size ± SD	*p* value
**WT**	+/+	3	217	25	8	8.7 ± 2.3	–
***Ccdc182***	−480/−480	3	193	23	8	8.4 ± 2.7	0.85
***Efcab15***	−/−	3	97	10	8	9.7 ± 1.5	0.34
***Fam187b***	−12225/−12225	3	222	26	8	8.5 ± 2.7	0.72
***Fam24a***	−2277/−2277	3	123	16	8	7.7 ± 3.1	0.55
***Fam24b***	−1214/−1214	3	249	29	8	8.6 ± 3.2	0.61
***Gfra2*♂**	−87933/−87933	3	138	16	8	8.6 ± 2.5	0.10
***Gfra2*♀**	−87933/−87933	8	194	25	10	7.8 ± 3.2	0.13
***Glipr1l1‐3***	−10138/−10138	5	320	47	8	6.8 ± 3.4	0.33
***Il3***	−1706/−1706	3	231	22	10	10.5 ± 2.0	0.19
***Izumo4***	−2398/−2398	3	227	27	8	8.4 ± 2.8	0.75
***Pin1rt1***	−528/−528	3	231	26	8	8.9 ± 1.5	0.55
***Slc22a16***	−33,732/−33,732	3	164	19	8	8.6 ± 2.3	0.82
***Spmip2***	−85,683/−85,683	3	207	22	8	9.4 ± 3.3	0.17
***Tex51***	−4231/−4231	4	279	33	8	8.5 ± 2.3	0.79
***Tmco2***	−3396/−3396	3	133	16	8	8.3 ± 3.3	0.81
***Triml1/Triml2***	−623,666/−623,666	3	169	23	8	7.3 ± 2.3	0.18

### 3.4 Phenotypic analyses of KO males

**FIGURE 2:**
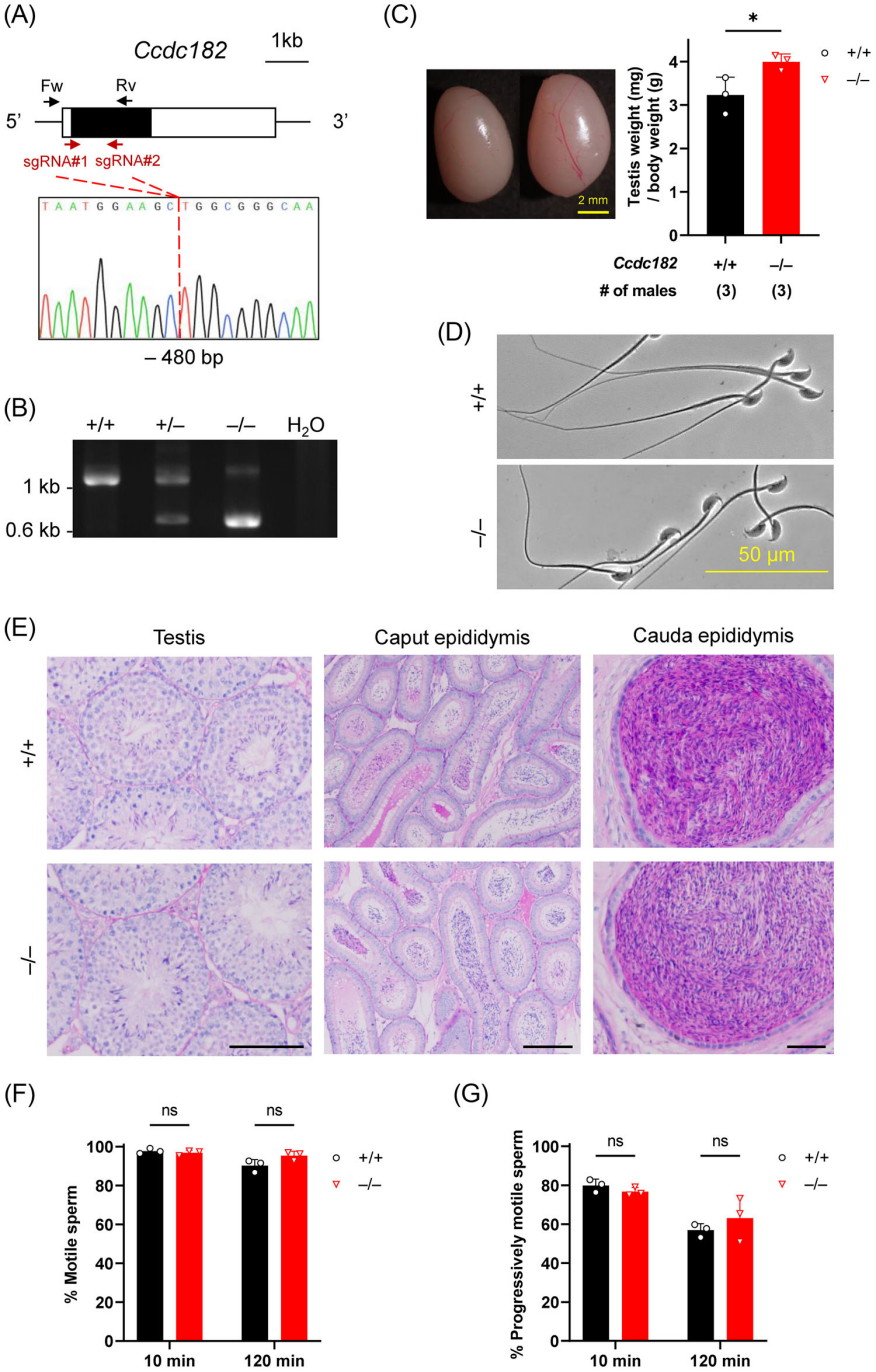
Phenotypic analysis of *Ccdc182* knockout (KO) male mice. (A) Genomic structure and KO strategy of mouse *Ccdc182*. To delete *Ccdc182*, sgRNA#1 and sgRNA#2 were employed to target its coding exon. Two primers (Fw, forward primer; Rv, reverse primer) flanking the truncated region were used to analyze the mouse genotype. The mutant sequence was cloned and analyzed by Sanger sequencing. (B) Genotypic validation of *Ccdc182* KO mice by polymerase chain reaction (PCR). The upper and lower bands represent the wild‐type (WT) and KO alleles, respectively. (C) Gross appearance and relative testis weight of WT and *Ccdc182* KO mice. Relative testis weight was calculated by dividing the testis weight (mg) by the corresponding body weight (g). (D) Sperm morphology of WT and *Ccdc182* KO males. (E) Histological analyses of testis, caput epididymis, and cauda epididymis in WT and *Ccdc182* KO males. Scale bars = 100 µm. (F) Motility of WT and *Ccdc182* KO spermatozoa. (G) Progressive motility of WT and *Ccdc182* KO spermatozoa.

**FIGURE 3:**
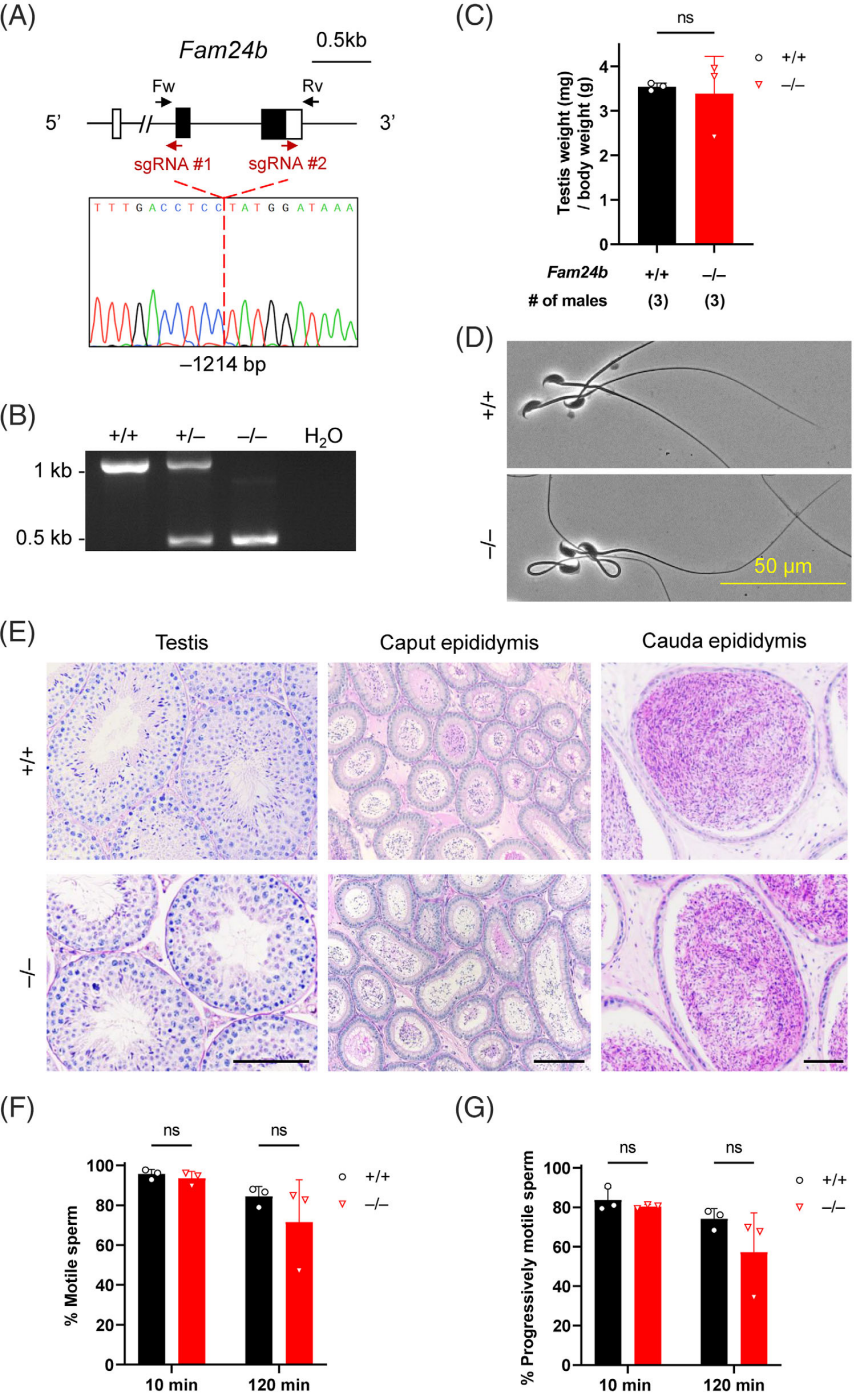
Phenotypic analysis of *Fam24b* knockout (KO) male mice. (A) Genomic structure and KO strategy of mouse *Fam24b*. To delete *Fam24b*, sgRNA#1 and sgRNA#2 were employed to target its coding exons. Two primers flanking the truncated region were used to analyze the mouse genotype. The mutant sequence was cloned and analyzed by Sanger sequencing. (B) Genotypic validation of *Fam24b* KO mice by polymerase chain reaction (PCR). The upper and lower bands represent the wild‐type (WT) and KO alleles, respectively. (C) Relative testis weight of WT and *Fam24b* KO mice. (D) Sperm morphology of WT and *Fam24b* KO males. (E) Histological analyses of testis, caput epididymis, and cauda epididymis in WT and *Fam24b* KO males. Scale bars = 100 µm. (F) Motility of WT and *Fam24b* KO spermatozoa. (G) Progressive motility of WT and *Fam24b* KO spermatozoa.

**FIGURE 4:**
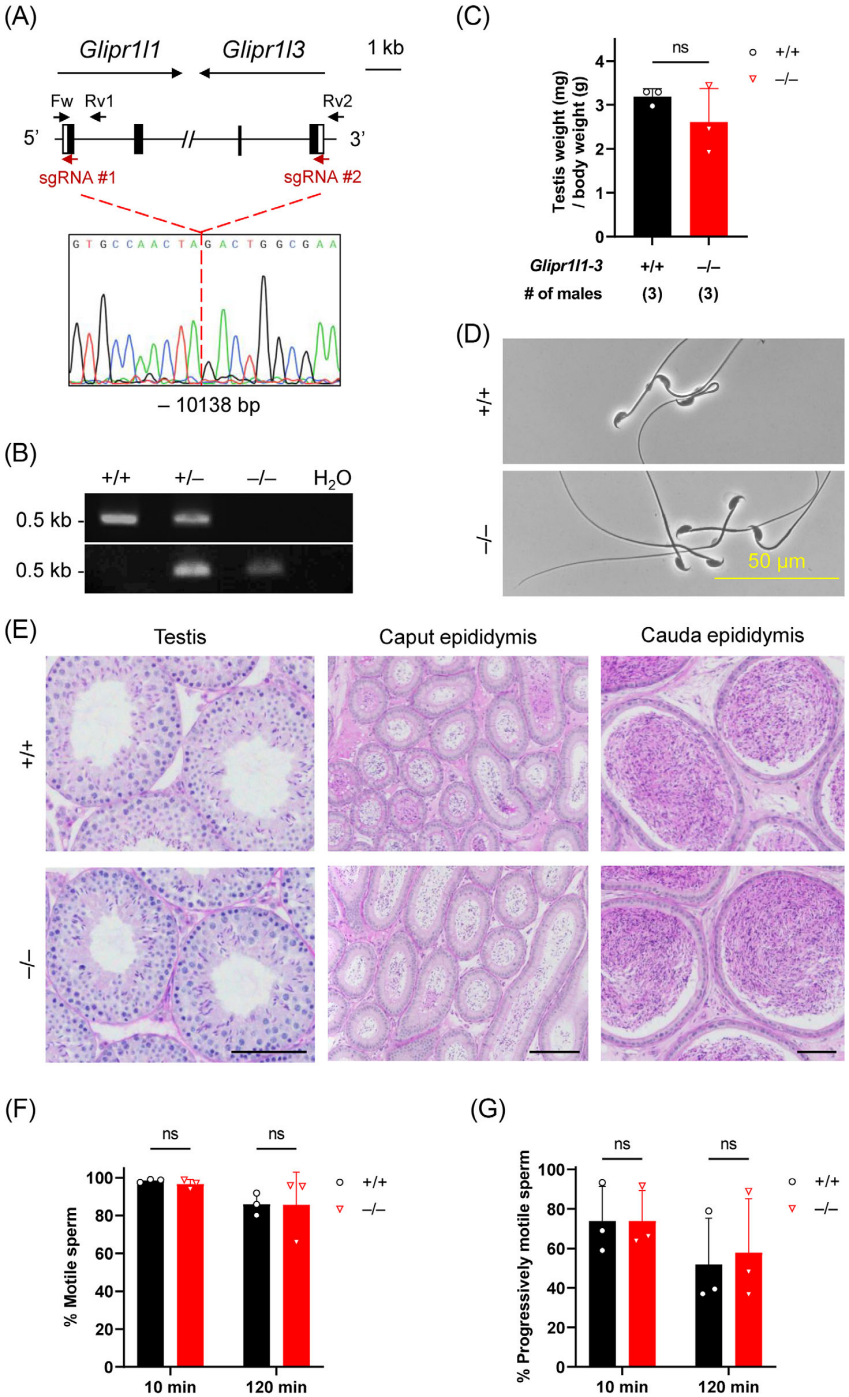
Phenotypic analysis of *Glipr1l1‐3* knockout (KO) male mice. (A) Genomic structure and KO strategy of mouse *Glipr1l1* and *Glipr1l3*. The direction of genes relative to the genome is indicated by arrows. *Glipr1l1* and *Glipr1l3* are, respectively, located at the forward and reverse strands of mouse chromosome 10. sgRNA#1 and sgRNA#2 were used to delete *Glipr1l1*, *Glipr1l2*, and *Glipr1l3*. Three primers (Fw, Rv1, and Rv2) were used to analyze the mouse genotype. The mutant sequence was cloned and analyzed by Sanger sequencing. (B) Genotypic validation of *Glipr1l1‐3* KO mice by polymerase chain reaction (PCR). The upper and lower bands represent wild‐type (WT) (amplified by Fw–Rv1) and KO alleles (amplified by Fw–Rv2), respectively. (C) Relative testis weight of WT and *Glipr1l1‐3* KO mice. (D) Sperm morphology of WT and *Glipr1l1‐3* KO males. (E) Histological analyses of testis, caput epididymis, and cauda epididymis in WT and *Glipr1l1‐3* KO males. Scale bars = 100 µm. (F) Motility of WT and *Glipr1l1‐3* KO spermatozoa. (G) Progressive motility of WT and *Glipr1l1‐3* KO spermatozoa.

**FIGURE 5:**
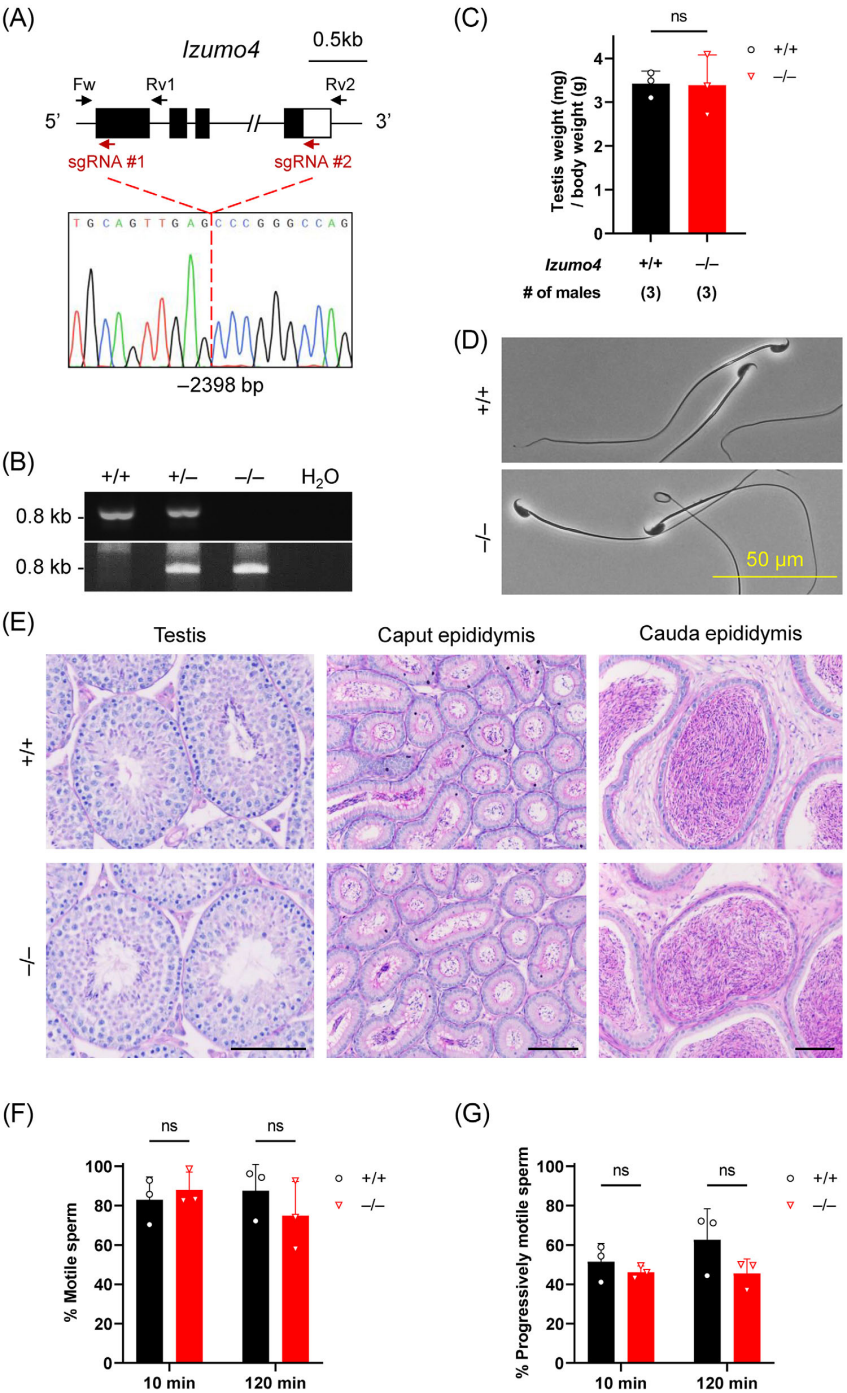
Phenotypic analysis of *Izumo4* knockout (KO) male mice. (A) Genomic structure and KO strategy of mouse *Izumo4*. sgRNA#1 and sgRNA#2 were employed to target its coding exons. Three primers (Fw, Rv1, and Rv2) were used to analyze the mouse genotype. The mutant sequence was cloned and analyzed by Sanger sequencing. (B) Genotypic validation of *Izumo4* KO mice by polymerase chain reaction (PCR). The upper and lower bands represent the wild‐type (WT; amplified by Fw1–Rv1) and KO alleles (amplified by Fw1–Rv2), respectively. (C) Relative testis weight of WT and *Izumo4* KO mice. (D) Sperm morphology of WT and *Izumo4* KO males. (E) Histological analyses of testis, caput epididymis, and cauda epididymis in WT and *Izumo4* KO males. Scale bars = 100 µm. (F) Motility of WT and *Izumo4* KO spermatozoa. (G) Progressive motility of WT and *Izumo4* KO spermatozoa.

**FIGURE 6:**
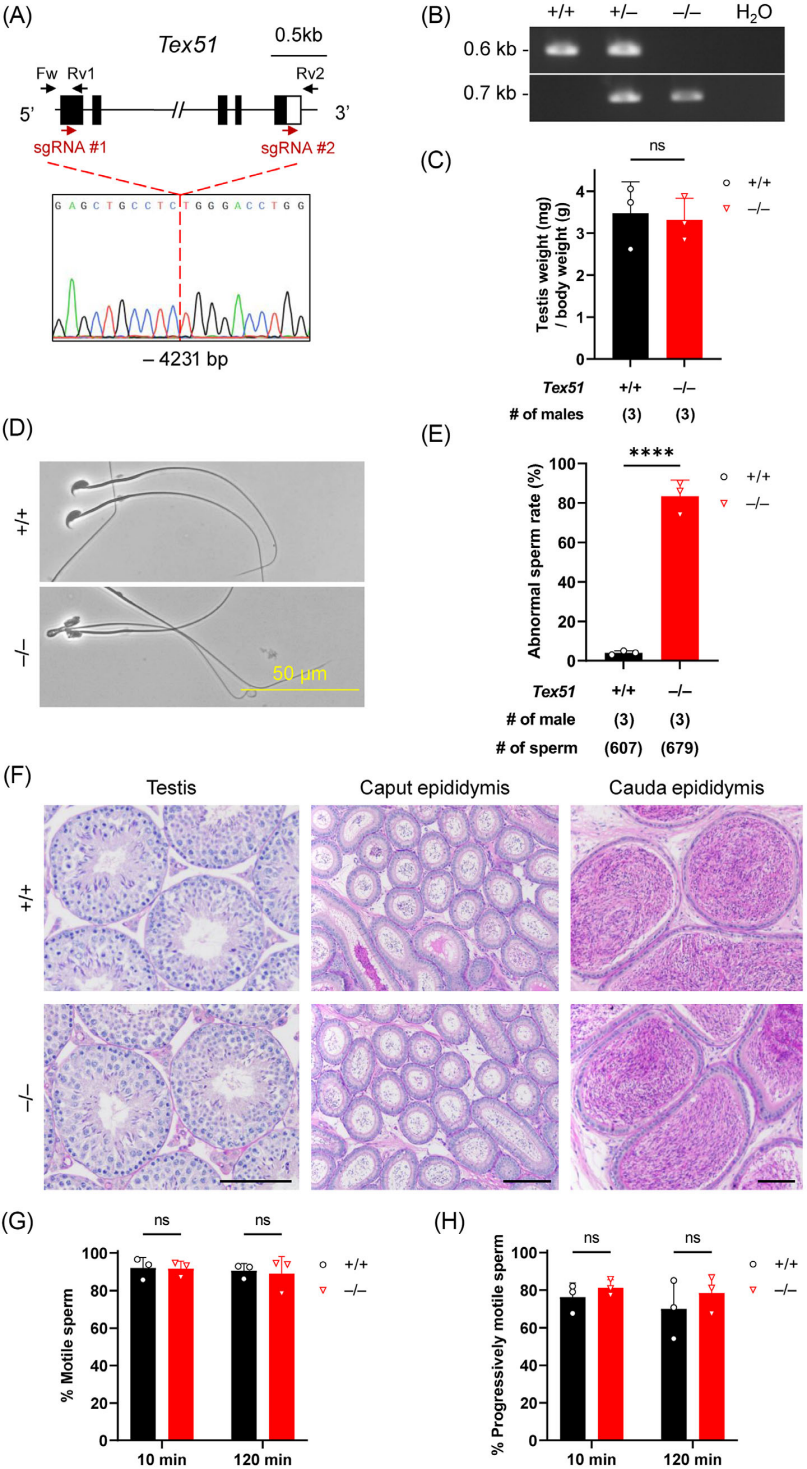
Phenotypic analysis of *Tex51* knockout (KO) male mice. (A) Genomic structure and KO strategy of mouse *Tex51*. To delete *Tex51*, sgRNA#1 and sgRNA#2 were employed to target its coding exons. Three primers (Fw, Rv1, and Rv2) were used to analyze the mouse genotype. (B) Genotypic validation of *Tex51* KO mice by polymerase chain reaction (PCR). The upper and lower bands represent the wild‐type (WT) (amplified by Fw1–Rv1) and KO alleles (amplified by Fw1–Rv2), respectively. (C) Relative testis weight of WT and *Tex51* KO mice. (D, E) Morphological analysis of WT and *Tex51* KO spermatozoa. (F) Histological analyses of testis, caput epididymis, and cauda epididymis in WT and *Tex51* KO males. Scale bars = 100 µm. (G) Motility of WT and *Tex51* KO spermatozoa. (H) Progressive motility of WT and *Tex51* KO spermatozoa.

Despite that the KO males exhibited normal fecundity, we conducted detailed analyses to rule out subtle impairments in their reproductive system that might not result in a detectable reduction in litter sizes. The phenotypic analyses of male mice lacking *Ccdc182* (Figures 2 and S6A), *Fam24b* (Figures 3 and S6B), *Glipr1l1‐3* (Figures 4 and S6C), *Izumo4* (Figures 5 and S6D), *Tex51* (Figures 6 and S6E) are described herein, whereas the analyses of *Fam187b*, *Pin1rt1*, *Slc22a16*, and *Tmco2* KO males are depicted in Figures S6F–H and S7–S10. Phenotypic analyses for *Efcab15*, *Fam24a*, *Gfra2*, *Il3*, *Spmip2*, and *Triml1/Triml2* KO mice were not conducted.

*Ccdc182* is a single‐exon gene located on the forward strand of mouse chromosome 11. To delete this gene, two sgRNAs targeting its coding exon and Cas9 were electroporated into WT zygotes (Figure 2A). The resultant 480 bp deletion in the *Ccdc182* locus was confirmed by genomic PCR and Sanger sequencing (Figure 2A,B). Notably, *Ccdc182* KO mice showed an increased relative testis weight compared with WT mice (Figure 2C). However, there were no apparent abnormalities in their sperm morphology (Figure 2D), testis and epididymis histology (Figure 2E), and sperm motility parameters (Figures 2F,G and S6A).

*Fam24b* is located on the reverse strand of mouse chromosome 7. By employing two sgRNAs targeting its two coding exons, we generated *Fam24b* KO mice carrying a 1214 bp deletion, which was confirmed by PCR and Sanger sequencing (Figure 3A,B). The KO males exhibited relative testis weight comparable to WT males (Figure 3C), as well as normal sperm morphology (Figure 3D) and testis and epididymis histology (Figure 3E). Computer‐aided sperm analysis revealed no significant differences in the motility, progressive motility, and swimming velocity between WT and *Fam24b* KO spermatozoa (Figures 3F,G and S6B).

*Glipr1l1*, *Glipr1l2*, and *Glipr1l3* are neighboring genes located on mouse chromosome 10 (Figure S11A). They encode proteins belonging to the glioma pathogenesis‐related 1 (GLIPR1) family and show homology in their amino acid sequences^40^, ^41^, ^42^ (Figure S11B). Two sgRNAs were designed to delete all three genes; a 10138 bp deletion was detected in the KO mice by genomic PCR and Sanger sequencing (Figure 4A,B). *Glipr1l1‐3* KO males exhibited no anomalies in their testis weight, testis and epididymis histology, or sperm morphology and motility (Figures 4C–G and S6C).

IZUMO4 is a secreted glycoprotein that belongs to the Izumo sperm–egg fusion family. By targeting its first exon and 3′ UTR, we generated KO mice with a 2398 bp deletion at the *Izumo4* locus (Figure 5A,B). The relative testis weight of KO mice was comparable to that of WT mice (Figure 5C). Furthermore, no obvious abnormalities were observed in the testis and epididymis histology, and sperm morphology, motility, and swimming velocity of the KO males (Figures 5D–G and S6D).

*Tex51* is located at the reverse strand of mouse chromosome 18. Similar to the structure of IZUMO1, TEX51 is predicted as a type‐I single‐pass transmembrane protein harboring a four‐helix bundle and a β‐hairpin at its ectodomain by the AlphaFold Protein Structure Database^43^ (AlphaFold DB Identifier: AF‐A0A140LIV7‐F1‐v4). A KO mouse line carrying a 4231 bp deletion was generated by the CRISPR/Cas9 system using two sgRNAs targeting the first coding exon and 3' UTR. The mutant allele was detected by genomic PCR and validated by Sanger sequencing (Figure 6A,B). Despite that *Tex51* KO males showed no overt abnormalities in their relative testis weight, testis and epididymis histology, and sperm motility, over 80% of the KO spermatozoa exhibited head malformations (Figures 6D–H and S6E).

Overall, our fertility tests and phenotypic analyses indicate that *Ccdc182*, *Efcab15*, *Fam187b*, *Fam24a*, *Fam24b*, *Gfra2*, *Il3*, *Izumo4*, *Pin1rt1*, *Slc22a16*, *Spmip2*, *Tex51*, and *Tmco2* are individually dispensable, whereas *Glipr1l1*, *Glipr1l2*, and *Glipr1l3*, as well as *Triml1* and *Triml2*, are collectively nonessential for male reproduction in mice. Notably, *Ccdc182* KO males displayed increased relative testis size and weight, and a portion of *Tex51* KO spermatozoa exhibited abnormal head morphogenesis. Although some subfertility indicators (e.g., aberrant sperm swimming trajectory, abnormal sperm morphology, reduced sperm count) might exist in several KO lines, subtle abnormalities did not negatively affect their fertility.

## 4 DISCUSSION

The male reproductive system expresses more than 1000 tissue‐specific genes, coordinating diverse and dynamic regulatory pathways across multiple cell types to support complex physiological processes such as testicular development, spermatogenesis, and hormone production.^44^, ^45^, ^46^ Despite their abundant expression in testis or epididymis, many of these genes have demonstrated dispensable for male fertility through mouse mutagenesis studies.^47^, ^48^, ^49^, ^50^ One proposed explanation of this phenomenon is transcriptional adaptation, in which the loss of a gene is compensated by the upregulation of other genes.^51^ Another hypothesis is transcriptional scanning, whereby the extensive transcription of genes safeguards the genome integrity of male germ cells.^52^ Although the depletion of such genes generally exhibits minimal impact on male fecundity under standard laboratory conditions, these genes may confer evolutionary advantages in the natural settings through some underexplored mechanisms such as sperm heteromorphism,^53^ sperm competition,^54^ or sexual selection.^55^

Among the 18 genes analyzed in this study, several are reportedly implicated in sperm functionality. GLIPR1L1 interacts with IZUMO1, an acrosomal membrane protein crucial for sperm–egg fusion, and localizes to the sperm acrosome. *Glipr1l1* KO spermatozoa exhibit a reduced ability to undergo the acrosome reaction and fertilize eggs in vitro^41^, ^42^; however, KO males sire pups with an average litter size comparable to WT males, suggesting that GLIPR1L1 alone is nonessential for male fertility. Driven by a hypothesis that the three GLIPR1‐like proteins are collectively indispensable for the acrosome reaction, we created a *Glipr1l1‐3* KO mouse line. Nevertheless, phenotypic analyses revealed that depletion of all three genes does not significantly affect male fertility (Figure 4).

Recent mouse mutagenesis analyses have shown that many testis‐enriched CCDC family proteins play pivotal roles in sperm formation, morphogenesis, and movement. CCDC38 interacts with CCDC42, intraflagellar transport protein 88 (IFT88), and outer dense fiber protein 2 (ODF2); ablation of CCDC38 impairs the flagellar formation and male fertility.^56^ CCDC181, a sperm flagellar protein, interacts with hook microtubule tethering protein 1 (HOOK1) in the manchette of early spermatids.^57^ *Ccdc181* KO male mice display reduced sperm counts, aberrant sperm head morphogenesis and flagellar formation, and impaired motility, collectively leading to male sterility. CCDC181 interacts with leucine‐rich repeat‐containing protein 46 (LRRC46), and its depletion diminishes the abundance of LRRC46.^58^ Notably, *Lrrc46* KO spermatozoa show morphological abnormalities resembling that of *Ccdc181* KO spermatozoa.^59^ CCDC189 is localized to the radial spoke of sperm axoneme and interacts with ciliary‐associated calcium‐binding coiled‐coil protein 1 (CABCOCO1). *Ccdc189* KO male mice show smaller testis weight, impaired spermiogenesis, and infertility, which phenocopies *Cabcoco1* KO mice.^60^, ^61^ Likewise, male patients carrying homozygous mutations in CCDC family proteins, such as CCDC9,^62^ CCDC28A,^63^, ^64^ CCDC65,^65^ CCDC155,^66^ CCDC157,^67^ and CCDC188,^68^, ^69^ are infertile due to impaired sperm motility and/or morphology. In this study, we revealed that disruption of *Ccdc182* solely does not compromise male fertility in mice; however, it remains possible that CCDC182 ensures normal sperm functioning in coordination with other CCDC proteins.

This study indicates that *Fam24a* and *Fam24b* are individually dispensable for male reproduction. The two genes encode proteins with high sequence homology (Figure S12A) and similar expression patterns in mouse spermatogenic cells (Figure 1A). Thus, it is tempting to speculate that these paralogous proteins compensate for each other in single KO mouse lines. Future investigation is warranted to determine whether FAM24A and FAM24B together play a significant role in male reproduction by creating a double KO mouse model.

*Pin1rt1* is annotated as a retrogene of *Pin1*, and the two genes encoding proteins with high sequence similarity (Figure S12B) and biased expression in mouse testis. While *Pin1rt1* is abundantly expressed in round spermatids, *Pin1* shows elevated expression in spermatogonia and Sertoli cells (Figure S12C). PIN1 regulates the architecture and function of serine/threonine (Ser/Thr)‐phosphorylated proteins by catalyzing the isomerization of Ser/Thr bonds preceding a proline (Pro) residue. PIN1 directly binds to a cell cycle regulator, cyclin D1, phosphorylated on its Thr‐286 succeeded by a proline.^70^ Truncation of *Pin1* in mice reduces the abundance of cyclin D1 and leads to defective proliferation of primordial germ cells, thus impairing both male and female fertility.^71^ We suspected that testis‐enriched PIN1RT1 may have a similar function given its sequence homology with PIN1. However, *Pin1rt1* KO males exhibit normal histology of seminiferous tubules and fertility (Figure S8), suggesting its dispensability in male reproduction.

## 5 SUMMARY

To permit precise diagnosis of male infertility, researchers have been exploring potential biomarkers using omics technologies.^72^, ^73^, ^74^ Proteomics, especially, has facilitated the high‐throughput identification of proteins with differential abundance in the seminal plasma of infertile men compared with healthy individuals. In this study, we discovered 18 genes dispensable for male reproduction. While these genes may have limited biological relevance, their enrichment in testis may make them potential biomarkers of sperm quality in men. Further clinical investigations are required to validate whether our observations in laboratory mice are applicable to male patients.

## AUTHOR CONTRIBUTIONS

Hsin‐Yi Chang, Yonggang Lu, and Masahito Ikawa designed the research and wrote the paper; Hsin‐Yi Chang, Yonggang Lu, Kaito Yamamoto, Jiang Sun, Keisuke Shimada, Yuki Hiradate, and Yoshitaka Fujihara performed experiments and analyzed the data.

## CONFLICT OF INTEREST STATEMENT

The authors declare no conflicts of interest.

## Supporting information



Supporting Information

Supporting Information
