# Supplementary material for: Mouse genome engineering uncovers 18 genes dispensable for male reproduction
Source: Andrology. 2025 Jun 26;14(3):833–46. doi: 10.1111/andr.70088 (PMC12917576; doi:10.1111/andr.70088)
Supplement: Supplementary file 2 — Supporting Information [file ANDR-14-833-s002.docx]

**Supplementary Tables**

**Table S1. KO Mouse lines generated in this study.** KO mouse lines have been deposited as frozen sperm to RIKEN BRC (RBRC) and CARD at Kumamoto University. The IDs for the lines that have yet to be deposited are marked as TBD (to be determined).

| **Gene symbol** | **Gene name** | **RBRC No.** | **CARD ID** |
| --- | --- | --- | --- |
| ***Ccdc182*** | Coiled-coil domain containing 182 | 12210 | 3441 |
| ***Efcab15*** | EF-hand calcium binding domain 15 | 4808 | N/A |
| ***Fam187b*** | Family with sequence similarity 187, member B | 12004 | 3373 |
| ***Fam24a*** | Family with sequence similarity 24, member A | 11475 | 3119 |
| ***Fam24b*** | Family with sequence similarity 24, member B | 11987 | 3356 |
| ***Gfra2*** | Glial cell line derived neurotrophic factor family receptor alpha 2 | TBD | TBD |
| ***Glipr1l1*** | GLI pathogenesis-related 1 like 1 | 12445 | 3544 |
| ***Glipr1l2*** | GLI pathogenesis-related 1 like 2 |  |  |
| ***Glipr1l3*** | GLI pathogenesis-related 1 like 3 |  |  |
| ***Il3*** | Interleukin 3 | 11023 | 2930 |
| ***Izumo4*** | IZUMO family member 4 | TBD | TBD |
| ***Pin1rt1*** | Peptidyl-prolyl cis/trans isomerase, NIMA-interacting 1, retrogene 1 | TBD | TBD |
| ***Slc22a16*** | Solute carrier family 22 (organic cation transporter), member 16 | 112211 | 3442 |
| ***Spmip2*** | Sperm microtubule inner protein 2 | 11977 | 3346 |
| ***Tex51*** | Testis expressed 51 | TBD | TBD |
| ***Tmco2*** | Transmembrane and coiled-coil domains 2 | 11995 | 3364 |
| ***Triml1*** | Tripartite motif family-like 1 | 11265 | 3069 |
| ***Triml2*** | Tripartite motif family-like 2 |  |  |

**Table S2. sgRNAs designed to target the 5' and 3' regions of the candidate genes.** The 20-bp nucleotides upstream (Up) and downstream (Down) of the truncated region are provided, with uppercase and lowercase indicating exon and intron, respectively.

| **Gene symbol** | | **sgRNAs** | | **Mutant sequence** | | **Deletion size (bp)** | |
| --- | --- | --- | --- | --- | --- | --- | --- |
| ***Ccdc182*** | | 5': GCCTGAAAGAGGGCTTCCAT  3': AGTGTGCGCCCGAGCTGGCG | | Up: cagaaggagataATGGAAGC  Down: TGGCGGGCAAGATTGTTTTT | | −480 | |
| **Fam187b** | | 5': GACAACTGCAGGTCGCTCCC  3': GATCAATAGGTAAGCCGAGC | | Up: GCCTGATGGGAGGGTACTGC  Down: cttacctattgatcaccccc | | −12,225 | |
| ***Fam24a*** | | 5': TGTGAAAGATGTTCGACTTG  3': CTGAAACCCCAAGCCTACGT | | Up: agGTGTGAAAGATGTTCGAC  Down: cgtaggccctttgtctgcca | | −2,277 | |
| ***Fam24b*** | | 5': TGATCGTGATTATTGTCCGG  3': GGACTCTGACATGGCACCTA | | Up: GTATAAGCCTTTTGACCTCC  Down: TATGGATAAAAGGAAATCTT | | −1,214 | |
| ***Gfra2*** | | 5': CGGGATCTCCGCATTGGATT  3': TCAGGCGGGAGTTCGTATCT | | Up: CGGCCGCCTGGCGGGATCTC  Down: tctctctctctctctctctc | | −87,933 | |
| ***Glipr1l1-3*** | | 5': AATTTCGGATCCGTGATAGT  3': AACACTTTCGCCAGTCTAAG | | Up: TCTTCCTAGGGTGCCAACTA  Down: gactgccttgaacactttcg | | −10,138 | |
| ***Il3*** | | 5': TGGTTAAACGGTGGGTATCC  3': AGACCACCTCAGCCCGCATC | | Up: GCTTCAATCAGTGGCCGGGA  Down: GCTCCGTCTCTCCTAACCGT | | −1,706 | |
| ***Izumo4*** | | 5': GTCGCACTGCAGGCAGCCCC  3': GCAACAGTGCAGTTGAGTCC | | Up: GACAGCGGCGCTGGCCCGGG  Down: ctcaactgcactgttgcctc | | −2,398 | |
| ***Pin1rt1*** | | 5': AGGTTACTCCTGAGCGAAG  3': GCAGCAGGAAGAGCGTCCAT | | Up: tatagGTTACTCCTGAGCGA  Down: GACGCTCTTCCTGCTGCTGT | | −528 | |
| ***Slc22a16*** | | 5': TGCAGGACTCCATGTCCGGG  3': TTTGGTTACAGTACAGACCG | | Up: AGGGCGCTCAGCACTGCCAC  Down: ccgtgggattgtcttgatta | | −33,732 | |
| ***Spmip2*** | | 5': ACACGGTCTTTGCCGTTTAC  3': TGTCACTGAGGGCCATGCGT | | Up: gagacacggtctttgccgtt  Down: catggccctcagtgacatca | | −85,683 | |
| ***Tex51*** | | 5': GGAAGAGCTGCCTCCGCTGC  3': TTGCACAAAGCACTCCTTCC | | Up: CCACTGGGAAGAGCTGCCTC  Down: tgggacctggagagctctga | | −4,231 | |
| ***Tmco2*** | | 5': TCACAAAGCACGGCTCATAA  3': GGAGAACAAAGTGCGAGACC | | Up: agtgacatcacaaagcacgg  Down: CCTGGAGGGGATAATCGTCG | | −3,396 | |
| ***Triml1/Triml2*** | | 5': GAGCAGCCTAGTCATTAACG  3': ATATTGGTTTAATCGCTCCT | | Up: ccaaggagcagcctagtcat  Down: TTTCTCTGACCTTACCTTCC | | −623,666 | |

**Table S3. The outcomes of embryo transfer and gene deletion.** The missing data is marked as ND (not determined).

| **Gene symbol** | **No. of pups delivered / No. of total embryos transplanted** (%) | **No. of KO pups obtained / No. of pups genotyped** (%) |
| --- | --- | --- |
| ***Ccdc182*** | 18/54 (33.3%) | 7/18 (38.9%) |
| ***Fam187b*** | 7/43 (16.3%) | 2/7 (28.6%) |
| ***Fam24a*** | 11/122 (9.0%) | 6/11 (54.5%) |
| ***Fam24b*** | 14/50 (28.0%) | 5/14 (35.7%) |
| ***Gfra2*** | ND | 2/6 (33.3%) |
| ***Glipr1l1-3*** | 17/53 (32.1%) | 3/17 (17.6%) |
| ***Il3*** | 18/95 (18.9%) | 8/18 (44.4%) |
| ***Izumo4*** | 10/58 (17.2%) | 3/10 (30%) |
| ***Pin1rt1*** | 1/55 (1.8%) | 1/1 (100%) |
| ***Slc22a16*** | 13/56 (23.2%) | 5/13 (38.5%) |
| ***Spmip2*** | 11/29 (37.9%) | 3/11 (27.3%) |
| ***Tex51*** | 22/60 (36.7%) | 4/22 (18.2%) |
| ***Tmco2*** | 16/45 (35.6%) | 8/16 (50%) |
| ***Triml1/Triml2*** | 35/98 (35.7%) | 3/35 (8.6%) |

**Table S4. Primer sets and PCR conditions for genotyping KO mice.**

| **Gene symbol** | **Primer sequences** | | **Annealing** | **Elongation** |
| --- | --- | --- | --- | --- |
|  | WT allele | KO allele |  |  |
| ***Ccdc182*** | Fw: CTCGGGGTTAAAGGAACGCT  Rv: CTCACTAAGCCCCCAACCTG | Fw: CTCGGGGTTAAAGGAACGCT  Rv: CTCACTAAGCCCCCAACCTG | 65 °C  30 s | 72 °C  120 s |
| ***Efcab15*** | Fw: CCCAATACTGTCCCCGCCTCTTCCTC  Rv: TTCTTTTGGCTCAGGTCCCACTCTAA | Fw: CCCAATACTGTCCCCGCCTCTTCCTC  Rv: TTCTTTTGGCTCAGGTCCCACTCTAA | 60 °C  30 s | 72 °C  30 s |
| ***Fam187b*** | Fw2: GCGCAAACGTCTAGGCTACT  Rv1: GGAAGAAGTGCTGGCTTGTC | Fw1: GTGGAGAAACTCGCTGAACC  Rv2: GCACCCCTCATTCTCCTGTA | 65 °C  30 s | 72 °C  30 s |
| ***Fam24a*** | Fw: AAGGGGTTTGGCTGGAAG  Rv: TCGGCACACATACAAACTCA | Fw: AAGGGGTTTGGCTGGAAG  Rv: TCGGCACACATACAAACTCA | 60 °C  30 s | 72 °C  30 s |
| ***Fam24b*** | Fw: TTTTATGCGGAAAACTTTCC  Rv: CTGAGGTTGGTCCTCGGTGG | Fw: TTTTATGCGGAAAACTTTCC  Rv: CTGAGGTTGGTCCTCGGTGG | 65 °C  30 s | 72 °C  90 s |
| ***Gfra2*** | Fw: GAAGTGCAGGGGAATTGGAC  Rv1: GAAGTCTCCTTCTCGGTCCT | Fw: GAAGTGCAGGGGAATTGGAC  Rv2: GCCTGCTTGTCTGTTTGGTT | 65 °C  30 s | 72 °C  30 s |
| ***Glipr1l1-3*** | Fw: TTCCCAGTCCAAGCACTCTC  Rv1: TCATCTCCCTCCCCTGCATA | Fw: TTCCCAGTCCAAGCACTCTC  Rv2: TACAGGGCTCTGACAATGGG | 65 °C  30 s | 72 °C  30 s |
| ***Il3*** | Fw: TAAAGCTGCTTCTGATGCCT  Rv: GGTAAAGCCAAAGGAGAGTTG | Fw: TAAAGCTGCTTCTGATGCCT  Rv: GGTAAAGCCAAAGGAGAGTTG | 65 °C  30 s | 72 °C  30 s |
| ***Izumo4*** | Fw: GACCAGAGTTGGAAGCATGA  Rv1: ATAGCTTCTTCCGGGCTAAG | Fw: GACCAGAGTTGGAAGCATGA  Rv2: TCTGTGGATAGGAGCGTAGG | 65 °C  30 s | 72 °C  50 s |
| ***Pin1rt1*** | Fw: CCCAGAATTTGGGAGACAGA  Rv: TGCATCTTGAGGCTGAGTTG | Fw: CCCAGAATTTGGGAGACAGA  Rv: TGCATCTTGAGGCTGAGTTG | 60 °C  30 s | 72 °C  60 s |
| ***Slc22a16*** | Fw2: GCTATTTGTTGAAAACGTGG  Rv1: TAAAGATAATGAGAACATGG | Fw1: AGTTGACGCACGTGCAATGG  Rv2: CAAATACTTGACACAGAACC | 65 °C  30 s | 72 °C  30 s |
| ***Spmip2*** | Fw1: GGATGCTGGTGACGAACTCA  Rv1: TTGGGACTGTGCAGCTACTG | Fw1: TGGAAACCGGAACTCAGAGC  Rv2: AGGAGGGTGTTCTCACTGGT | 65 °C  30 s | 72 °C  30 s |
| ***Tex51*** | Fw: GTCTGCACCAGTAGCAAGGA  Rv1: CCAGGGCAAGAATATGGTGT | Fw: GTCTGCACCAGTAGCAAGGA  Rv2: GGACACCAGCCTAGGTGATT | 65 °C  30 s | 72 °C  50 s |
| ***Tmco2*** | Fw: CCCACTTCTAGTGATATTGG  Rv1: TCCAATTGCCAACAAAGTCA | Fw: CCCACTTCTAGTGATATTGG  Rv2: TATCTTGCATTACCATGAGG | 60 °C  30 s | 72 °C  30 s |
| ***Triml1/Triml2*** | Fw2: CTGGAGAGCTGGAGCGTATG  Rv1: GAAACACGCCTTGTTCGTCC | Fw1: GAGCCCTAAGCCTCCTCTGGAAAGC  Rv2: GAGAGCCACATTAAGTCTAATGGCTT | 65 °C  30 s | 72 °C  30 s |
